# A Virtual Book Club for Professional Development in Emergency Medicine

**DOI:** 10.5811/westjem.2020.11.49066

**Published:** 2020-12-14

**Authors:** Jaime Jordan, Rebecca A. Bavolek, Pamela L. Dyne, Chase E. Richard, Stephen Villa, Natasha Wheaton

**Affiliations:** University of California, Los Angeles, David Geffen School of Medicine, Department of Emergency Medicine, Los Angeles, California

## Abstract

**Introduction:**

Professional development is an important component of graduate medical education, but it is unclear how to best deliver this instruction. Book clubs have been used outside of medicine as a professional development tool. We sought to create and evaluate a virtual professional development book club for emergency medicine interns.

**Methods:**

We designed and implemented a virtual professional development book club during intern orientation. Afterward, participants completed an evaluative survey consisting of Likert and free-response items. Descriptive statistics were reported. We analyzed free-response data using a thematic approach.

**Results:**

Of 15 interns who participated in the book club, 12 (80%) completed the evaluative survey. Most (10/12; 83.3%) agreed or strongly agreed that the book club showed them the importance of professional development as a component of residency training and helped them reflect on their own professional (11/12; 91.7%) and personal development (11/12; 91.7%). Participants felt the book club contributed to bonding with their peers (9/12; 75%) and engagement with the residency program (9/12; 75%). Our qualitative analysis revealed five major themes regarding how the book club contributed to professional and personal development: alignment with developmental stage; deliberate practice; self-reflection; strategies to address challenges; and communication skills.

**Conclusion:**

A virtual book club was feasible to implement. Participants identified multiple ways the book club positively contributed to their professional development. These results may inform the development of other book clubs in graduate medical education.

## BACKGROUND

Professional development, defined by the US Centers for Disease Control and Prevention as “a systematic process that strengthens how professionals obtain and retain knowledge, skills and attitudes,” is essential for trainees in order to meet the challenges of medical practice and develop into physician leaders.^1^ Professional development can help engage trainees in reflective and deliberate practice, allowing them to be better prepared for their future careers.^2^ It also serves to encourage the growth of physician advocates and leaders to meet the ever-changing, complex needs of the field of medicine.^3^ These demands require physicians to be capable of conducting self-directed development of their clinical competency, interpersonal dynamics, and overall professionalism. By improving engagement and continuing to develop the skill set required to meet the challenges of medical practice, professional development can help mitigate burnout and has been identified as a strategy for resilience in medicine.^4^–^5^

Similarly, personal development is also a lifelong learning process that incorporates self-reflection and external feedback to promote awareness of identity, achievement of goals, and enhanced quality of life.^6^ Personal and professional development are often intertwined in medicine, and both are important for medical trainees.^6^–^10^ The importance of professional development in emergency medicine (EM) residency education is supported by the regulatory requirements of the Accreditation Council for Graduate Medical Education (ACGME).^11^

Professional and personal development are complex constructs with little data to guide optimal teaching modalities. However, randomized controlled trials have revealed that teachers who received direct developmental interventions received higher overall teaching quality scores.^12^ Professional development opportunities abound in other business sectors using a wide spectrum of experiences, varying from online forums and longitudinal courses to conferences, mentoring groups, and case studies as well as book clubs.^13^–^14^ Book clubs have been used in other industries including business and teaching as a professional development tool that encourages active engagement, self-reflection, and team building.^15^–^16^

Book clubs have also been used for longitudinal professional development in other allied health professions and as a way to improve interprofessional communication, leadership skills, and learner’s understanding of the patient perspective.^17^–^22^ However, there is limited literature regarding the use of book clubs in graduate medical education (GME).^21^, ^23^–^24^ The literature that does exist primarily consists of curricular descriptions and limited outcome data, which have generally been positive.^21^, ^23^–^24^ As an example, Kan et al describe a book club for psychiatric trainees consisting of 90-minute, bimonthly sessions incorporating trainee led, instructor facilitated, in-depth discussions of nonfiction book content and application to psychiatric and clinical practice, which was felt by participants to positively contribute to training.^23^

## OBJECTIVES

The emergence of the COVID-19 pandemic has led to the transition of many medical education experiences to the virtual environment.^25^ This transition has created an opportunity for educators to develop remote learning methods to ensure high quality education at the GME level.^26^–^27^ To meet the demands of professional development education in a virtual setting we sought to create and evaluate a virtual professional development book club for EM interns. The goals of the book club were as follows: 1) to introduce residents to the importance of personal and professional development as a component of residency training; 2) to encourage the use of personal and professional development materials outside of medicine; and 3) to foster a culture of metacognition.

## CURRICULAR DESIGN

Our study team of medical educators designed the book club with input from our resident team member. We purposefully identified a broad range of books that addressed professional development topics based on national bestseller lists, book reviews, and the authors’ prior experiences with other professional development book clubs. We selected the final list of five books by group consensus (Appendix A). We chose to allow learners to select a book from our suggested list to augment learner agency and engagement. The use of multiple books also allowed the group to learn from each other as each book was discussed during the session. We planned the book club to be conducted over two hours and include an introduction, small-group breakout discussions on individual books, report out in a large group discussion, and summary and reflection of impact on learners as physicians and trainees. The book club director (NW) created the discussion questions based on the goals of the sessions with input from the study team. Open-ended questions were used to maximize depth of response and promote discussion. Discussion questions are available in Appendix B.

**Population Health Research Capsule**

What do we already know about this issue?

*Professional development is an important component of graduate medical education*,

but it is unclear how to best deliver this instruction.

What was the research question?

Can a virtual professional development book club be successfully implemented for EM interns?

What was the major finding of the study?

A virtual professional development book club was feasible to implement and perceived to be valuable.

How does this improve population health?

These results may inform the development of other book clubs in graduate medical education.

We contacted all 15 incoming EM interns one month prior to intern orientation and invited them to participate in the book club. In an effort to promote self-reflection and inquiry, we provided them the book list with a short description of each book and asked that they rank their book preferences. To ensure equal distribution of books, the book club director then assigned participants a book according to their preferences. All participants received their first or second choice. Participants read their book and participated in the virtual book club as outlined above. We implemented the book club, using the Zoom platform (Zoom Video Communications Inc., San Jose, CA) in June 2020 during intern orientation and prior to any clinical experiences.^28^ We chose Zoom as our virtual platform as this was already being used by the residency program for virtual didactics and all the faculty facilitators were familiar with the format. Faculty facilitators read the books, were oriented to the goals of the session, and moderated all discussions. The major resource requirements for the book club were faculty time and a virtual platform.

After the book club, we invited participants by email to complete a confidential online evaluative survey. We did not find any existing assessment tools that were appropriate for our context and setting during our literature review. Therefore, one author with advanced training in evaluation and survey design (JJ) developed an evaluative survey, incorporating established guidelines for survey research.^29^ The survey developer reviewed the literature and gathered input from the study team to maximize content validity. Building off of other book club evaluative instruments in the literature, consisting of agreement survey items and verbal feedback, we chose to include Likert agreement and free-response items in our evaluative survey.^17^,^22^,^23^ We read the survey aloud with study team members and piloted with a small group of reference subjects prior to implementation to optimize response process validity. We revised the survey for clarity based on feedback from piloting. The final version of the survey is available in Appendix C.

The study was deemed exempt by the Institutional Review Board of the David Geffen School of Medicine.

## IMPACT/EFFECTIVENESS

To assess the impact of our book club, we calculated and reported descriptive statistics for survey items with discrete answer choices. For free-response data, we performed a thematic qualitative analysis. Two researchers experienced in qualitative methods (JJ and SV) independently analyzed free-response data line by line to identify recurring concepts and assign codes that were further refined into themes using the constant comparative method.^30^ After initial independent review, the two analysts met to review codes and establish a final coding scheme. The two analysts then independently re-coded all data using this final coding scheme. Subsequently, the two analysts met again to discuss their findings and establish their agreement. The overall percent agreement between the analysts for the second round of coding was 91.7%. During this second meeting the analysts resolved discrepancies by in-depth discussion and negotiated consensus.

**Figure 1:**
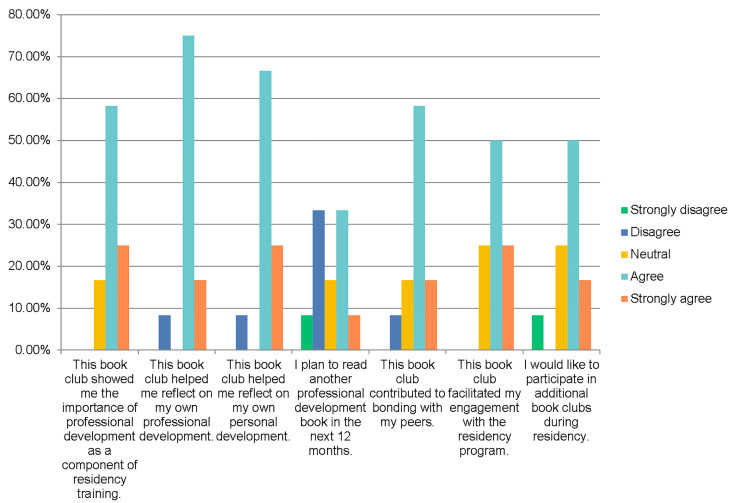
Participant perspectives of virtual book club.

All 15 interns in the incoming class participated in the book club and 12 (80%) completed the evaluative survey. Participant perspectives are displayed in Figure 1 and Table 1. Most (10/12; 83.3%) agreed or strongly agreed that the book club showed them the importance of professional development as a component of residency training. The majority of participants felt the book club helped them reflect on their own professional (11/12; 91.7%) and personal development (11/12; 91.7%). Participants also noted that the book club contributed to bonding with their peers (9/12; 75%) and engagement with the residency program (9/12; 75%). Two thirds of participants said they would like to participate in additional book clubs during residency. A minority (5/12; 41.7%) of participants planned to read another professional development book in the next 12 months.

Results of qualitative analysis are displayed in Table 2. Our qualitative analysis revealed five major themes regarding how the book club contributed to professional and personal development: alignment with developmental stage; deliberate practice; self-reflection; strategies to address challenges; and communication skills. Participants noted that the book club was also valuable in helping them engage with the program’s faculty and residents. Participants noted they planned to directly apply content discussed in the session. For example, related to the discussion on growth mindset, one participant remarked, “[I plan to] have a positive outlook and prepare to learn and grow throughout residency.” The one theme that emerged for improvement of the book club was the suggestion to hold it in an in-person setting.

GME training programs in EM require learners to simultaneously learn the core content of EM as well as develop the personal and professional skills necessary to safely navigate the multifaceted milieu of the emergency department (ED), an environment with multiple types of workers including nurses, technicians, clerical staff, and physician colleagues. Emergency physicians must develop robust professional skills to effectively interact with and lead multidisciplinary teams as well as to mitigate such factors as burnout in order to sustain a successful career in medicine. As opposed to procedural skills, medical knowledge, and concrete aspects of patient care, professionalism and communication-based competencies are relatively intangible and difficult to “teach.”

A professional development book club such as the one developed and evaluated in this study provides a potential strategy for developing these skills. While there is literature describing the importance of humanities in medical education and utilization of a book club experience in GME programs, we believe this is the first such experience to incorporate professional development books as the substrate for learning and discussion.^23^,^31^,^32^ Our results demonstrate that this type of educational offering was feasible to implement, well received by trainees, and has the potential to support trainees in self-reflection, deliberate practice, communication, and addressing challenges. Additionally, while we did not directly measure metacognition, the free-response data from participants indicated metacognitive activities such as planning an approach to learning, self-assessment and correction, and using appropriate strategies to solve a problem.

Participants felt this book club positively contributed to their professional development in several ways including the encouragement of deliberate practice, which has been identified in the literature as a key component in the development of expertise.^33^–^35^ Fostering this practice early in training may compound results. Participants also noted the book club promoted self-reflection, which has been described as being essential for improvement.^36^–^37^ Additionally, participants felt this session contributed to their development by introducing specific, actionable strategies to address challenges and build communication skills. The strategies introduced may be particularly beneficial in the ED where the ability to handle the unpredictability and critical nature of disease presentations, endure frequent interruptions, manage conflict, and use a team approach to patient care is crucial.^38^–^42^ Finally, both quantitative and qualitative results demonstrate that participants viewed this experience as an opportunity to engage with faculty and peers. Holding such an event early in training may help to more effectively and efficiently integrate new interns into the residency program. Forming bonds with faculty and peers can enable a strong support network and foster community, which can decrease burnout.^43^–^45^

The majority of participants felt that the session helped them think about how to deploy new professional skills as they entered into residency training. Given the perceived utility of the exercise, it was surprising that fewer than half of the cohort indicated that they planned to read another personal or professional development book in the coming 12 months. This most likely reflects their preconceived thoughts that they will need to spend what little time they have as interns reading medical content rather than professional development books. This finding emphasizes that the period of time prior to starting clinical rotations was the ideal time to hold this exercise in order to introduce the importance of professional development on EM practice. It may also be beneficial to incorporate such a session during the final months of medical school when student responsibilities have waned, as a deliberate attempt to jump start their subsequent postgraduate development.

We believe that it is our role as educators to ensure that our trainees are receiving a comprehensive medical education including EM core content and professional development. Deliberate attempts by program leadership to incorporate professional development into the curriculum may be necessary to ensure that these important topics are addressed. The majority of participants reported that they would like to participate in additional book clubs during residency. An in-person setting was recommended for improvement, which likely reflects the less personal nature of the virtual environment. Although the book club experience would likely have been richer as an in-person event, the participants’ experiences with this version showed that value was maintained using a virtual platform.

We developed this book club as a novel experience to encourage our interns to embark on a path of personal and professional development from the very beginning of residency training. We also hope this experience fosters an awareness of the opportunities to develop these less tangible skills in the clinical environment, as well as the benefit of reaching outside of the medical sphere for expertise in skill development. It is our hope that an increased knowledge of variances in mindset, learning style, negotiation tactics, and communication skills will allow our interns to observe, reflect on, and model others’ behaviors as they progress through their training. In the future we plan to evaluate objective learning outcomes, as these were not assessed in the current study, and to expand the scope of a professional development-themed book club.

## LIMITATIONS

There are several limitations that must be considered. This study was conducted at a single academic institution so the results may not be generalizable. Additionally, the sample size was small and only consisted of interns; thus, it is unclear whether these same results would be found across postgraduate year levels. We believe these limitations are acceptable as an initial assessment of a novel educational session. Although the response rate was good, it is possible that non-responders may have answered differently than those who completed the evaluative survey. Additionally, while the survey was confidential and it was explicitly communicated to participants that we were interested in honest and candid feedback as this was a new educational session, there is the possibility of response bias. Finally, based upon feedback, holding this session in person may have been beneficial and it is unclear how the setting influenced the impact of the book club.

## CONCLUSION

In summary, a virtual book club was feasible to implement and participants perceived positive contributions to their professional development. Potential positive outcomes include encouragement of deliberate practice and self-reflection, improved communication skills and strategies to address challenges, and engagement with each other as well as residency program leadership. These results may inform the development of other book clubs in graduate medical education.

## Supplementary Information







## Figures and Tables

**Table 1:** Participant perspectives of virtual book club.

	Strongly disagree n (%)	Disagree n (%)	Neutral n (%)	Agree n (%)	Strongly agree n (%)	Total n (%)
This book club showed me the importance of professional development as a component of residency training.	0 (0)	0 (0)	2 (16.7)	7 (58.3)	3 (25.0)	12 (100%)
This book club helped me reflect on my own professional development.	0 (0)	1 (8.3)	0 (0)	9 (75.0)	2 (16.7)	12 (100%)
This book club helped me reflect on my own personal development.	0 (0)	1 (8.3)	0 (0)	8 (66.7)	3 (25.0)	12 (100%)
I plan to read another professional development book in the next 12 months.	1 (8.3)	4 (33.3)	2 (16.7)	4 (33.3)	1 (8.3)	12 (100%)
This book club contributed to bonding with my peers.	0 (0)	1 (8.3)	2 (16.7)	7 (58.3)	2 (16.7)	12 (100%)
This book club facilitated my engagement with the residency program.	0 (0)	0 (0)	3 (25.0)	6 (50.0)	3 (25.0)	12 (100%)
I would like to participate in additional book clubs during residency.	1 (8.3)	0 (0)	3 (25.0)	6 (50.0)	2 (16.7)	12 (100%)

**Table 2:** Results of qualitative analysis of interns’ perceptions regarding the use of a virtual book club for professional development.

Domain	Major themes	Exemplar quotes
Contribution to professional and personal development	Alignment with developmental stage	“This book was appropriate for someone about to start residency.”
	Deliberate practice	“Applying a more intentional perspective towards learning in a meaningful manner in residency.” “…thinking more about how to organize my goals in relation to my overall purpose. I believe this will be helpful in terms of prioritizing where I direct my energy.”
	Self-reflection	“Insight into how my professional development has been influenced by my personal characteristics.” “It helped me reflect on my own tendencies in difficult interactions and how those can be improved.”
	Strategies to address challenges	“The book made me reflect on how to deal with challenges in life and how to persist and use the opportunity to grow.”
	Communication skills	“[This book club] improved my communication skills.”
Additional value of book club	Opportunity to engage with program faculty	“I enjoyed getting to know the faculty better in small groups.”
	Opportunity to engage with program residents	“I appreciated participating with my peers.”
Plans for change after book club	Direct application of discussed content	“I will try and take a step back and remove my emotion from the situation when faced with a difficult interaction.”
Improvement of book club	In-person setting	“I think this was a great idea, however, I think this would be much better in person.”
