# Supplementary material for: A Virtual Book Club for Professional Development in Emergency Medicine
Source: West J Emerg Med. 2020 Dec 14;22(1):108–14. doi: 10.5811/westjem.2020.11.49066 (PMC7806317; doi:10.5811/westjem.2020.11.49066)
Supplement: Supplementary file 1 [file wjem-22-108-s001.docx]

**Appendix A. Book List**

1. Voss, C and T. Raz. **Never Split the Difference: Negotiating as if your life depends on it.** First Edition. HarperCollins Publishers. 2016.

2. Stone, D and S. Heen. **Thanks for the Feedback: The Science and Art of Receiving Feedback Well.** First Edition. Viking. 2014.

3. Ury W. **Getting Past No:  Negotiating in Difficult Situations**. Revised Edition. Bantam. 1993.

4. Dweck C. **Mindset: The New Psychology of Success**. Updated Edition. Ballantine Books. 2007.

5. Duckworth A. **Grit: The Power of Passion and Perseverance**. Scribner. Reprinted Edition. 2018.
