# Supplementary material for: A Virtual Book Club for Professional Development in Emergency Medicine
Source: West J Emerg Med. 2020 Dec 14;22(1):108–14. doi: 10.5811/westjem.2020.11.49066 (PMC7806317; doi:10.5811/westjem.2020.11.49066)
Supplement: Supplementary file 2 [file wjem-22-108-s002.docx]

**Appendix B. Book club discussion questions**

**Small group discussion questions**

1. What drives human behavior? What drives our behavior as physicians? What drives our patients' behavior? How did this book make you reflect on your own behavior? How can you use the things you learned in the book to affect the behavior of those around you?
2. What did you learn about communication in this book? How might it impact how you communicate in the ED (content, tone, body language etc.)?
3. What pitfalls regarding how we think (e.g. bias) did you learn about in this book? Which are you as an individual particularly vulnerable to? What about the ED do you think either protects us or makes us more vulnerable to these pitfalls?
4. What are the main take-home points from this book that will affect your behavior in the ED? In life outside the ED? How will you use the lessons in this book to impact your own professional and/or personal development?
5. Are there any other specific questions this book raised you want to discuss?

**Large group discussion questions**

1. What are the top three take home lessons you learned from this book that are applicable to the emergency department and your professional development as an emergency medicine physician?
2. What lessons did you learn from this book that may be applicable to your life outside of the emergency department and your personal development?
3. Was there anything in this book that did not resonate with you or other group members?
