# Supplementary material for: A Virtual Book Club for Professional Development in Emergency Medicine
Source: West J Emerg Med. 2020 Dec 14;22(1):108–14. doi: 10.5811/westjem.2020.11.49066 (PMC7806317; doi:10.5811/westjem.2020.11.49066)
Supplement: Supplementary file 3 [file wjem-22-108-s003.docx]

**Appendix C. Book Club Evaluation**

Please rate your agreement with the following statements:

1. This session showed me the importance of professional development as a component of residency training.

Strongly disagree         disagree           neutral             agree                strongly agree

2.  This session helped me reflect on my own professional development.

Strongly disagree         disagree           neutral             agree                strongly agree

3.  This session helped me reflect on my own personal development.

Strongly disagree         disagree           neutral             agree                strongly agree

4.  I plan to read another professional development book in the next 12 months.

Strongly disagree         disagree           neutral             agree                strongly agree

5.  This session contributed to bonding with my peers.

Strongly disagree         disagree           neutral             agree                strongly agree

6.  This session facilitated my engagement with the residency program.

Strongly disagree         disagree           neutral             agree                strongly agree

7.  I would like to participate in additional book club sessions during residency.

Strongly disagree         disagree           neutral             agree                strongly agree

8.  In what ways did this session contribute to your professional development?

9.  In what ways did this session contribute to your personal development?

10.  Please describe any other ways the book club was valuable to you:

11. What is one thing you are going to implement or do differently after this book club?

12.  Please provide any suggestions for improvement of this book club:
